# Lessons learned from pre-implementation activities to integrate a web-based personalized health risk assessment program in diverse primary care settings

**DOI:** 10.1186/1748-5908-10-S1-A63

**Published:** 2015-08-20

**Authors:** Lori A Orlando, R Ryanne Wu, Nina Sperber, Geoffrey S Ginsburg, Corrine Voils

**Affiliations:** 1 Department of Medicine, Duke University, Durham, NC 27705, USA; 2 Center for Applied Genomics and Precision Medicine, Duke University, Durham, NC 27705, USA; 3 Department of Health Services Research, Department of VA Affairs, Durham, NC 27705, USA

## Introduction

Risk assessment, which can target preventive care to individuals' disease risk, is becoming an increasingly important strategy for improving population health. However, numerous barriers limit the collection and synthesis of the data necessary for risk-stratification. Leveraging health IT may prove critical in overcoming these barriers and improving uptake of preventive guidelines. To this end, NHGRI is funding implementation of MeTree, a patient-facing health risk assessment program with just-in-time education/clinical decision support, into 5 diverse U.S. healthcare systems. We developed a mixed-methods pre-implementation process to facilitate uptake of MeTree that highlights implementation support needs. Here we present findings from 2 of the 5 systems.

## Methods/Results

Our pre-implementation framework components include: (1) web-based provider educational tools, (2) identification of a clinic champion at each clinic, (3) site visits for information gathering and education, (4) administration of Organizational Readiness for Implementing Change (ORIC) survey to all providers/staff, and (5) semi-structured interviews with providers/staff addressing ORIC domains. Descriptive statistics characterized organizational readiness in each healthcare system and ICCs agreement among providers/staff. Qualitative data, analyzed using ORIC domains and data-derived categories, were integrated with quantitative findings. Results: There was poor within-clinic agreement about organizational readiness to change (ICC = 0.05-0.22), although 76% (N-26/34) somewhat agree/agree that their clinic was committed to implementing MeTree. Many were unclear about integration steps (e.g., 35% (N = 12/34) disagree/somewhat disagree that their clinic knows the steps involved in implementation). Qualitative data revealed concern about impact on workflow and physician-patient communication.

## Conclusion

Providers and staff value health IT to support risk assessment with just-in-time education. However, promotion via clinical champions and education is insufficient for successful implementation within busy primary care clinics. Key concerns that must be addressed include work-flow and communication during provider appointments. Identifying other promotion processes with/without a clinical champion should be further explored.

